# Vascular plants promote ancient peatland carbon loss with climate warming

**DOI:** 10.1111/gcb.13213

**Published:** 2016-03-08

**Authors:** Tom N. Walker, Mark H. Garnett, Susan E. Ward, Simon Oakley, Richard D. Bardgett, Nicholas J. Ostle

**Affiliations:** ^1^ Faculty of Life SciencesThe University of ManchesterMichael Smith BuildingOxford RoadManchesterM13 9PTUK; ^2^ Lancaster Environment CentreLancaster UniversityBailriggLancasterLA1 4YQUK; ^3^ Centre for Ecology and HydrologyLancaster Environment CentreLibrary AvenueBailriggLancasterLA1 4APUK; ^4^ NERC Radiocarbon FacilityScottish Enterprise Technology ParkRankine AvenueEast KilbrideGlasgowG75 0QFUK

**Keywords:** climate warming, dwarf‐shrubs, ecosystem respiration, graminoids, peatlands, priming, radiocarbon, vegetation change

## Abstract

Northern peatlands have accumulated one third of the Earth's soil carbon stock since the last Ice Age. Rapid warming across northern biomes threatens to accelerate rates of peatland ecosystem respiration. Despite compensatory increases in net primary production, greater ecosystem respiration could signal the release of ancient, century‐ to millennia‐old carbon from the peatland organic matter stock. Warming has already been shown to promote ancient peatland carbon release, but, despite the key role of vegetation in carbon dynamics, little is known about how plants influence the source of peatland ecosystem respiration. Here, we address this issue using *in situ* ^14^C measurements of ecosystem respiration on an established peatland warming and vegetation manipulation experiment. Results show that warming of approximately 1 °C promotes respiration of ancient peatland carbon (up to 2100 years old) when dwarf‐shrubs or graminoids are present, an effect not observed when only bryophytes are present. We demonstrate that warming likely promotes ancient peatland carbon release *via* its control over organic inputs from vascular plants. Our findings suggest that dwarf‐shrubs and graminoids prime microbial decomposition of previously ‘locked‐up’ organic matter from potentially deep in the peat profile, facilitating liberation of ancient carbon as CO_2_. Furthermore, such plant‐induced peat respiration could contribute up to 40% of ecosystem CO_2_ emissions. If consistent across other subarctic and arctic ecosystems, this represents a considerable fraction of ecosystem respiration that is currently not acknowledged by global carbon cycle models. Ultimately, greater contribution of ancient carbon to ecosystem respiration may signal the loss of a previously stable peatland carbon pool, creating potential feedbacks to future climate change.

## Introduction

Ecosystem respiration is the largest land to atmosphere carbon dioxide (CO_2_) flux, accounting for more than half of all biospheric CO_2_ emissions (IPCC, 2013). Climate warming is expected to increase ecosystem respiration globally (Davidson & Janssens, 2006; IPCC, 2013), but the magnitude of its impact will depend on additional factors that may themselves be temperature dependent (Davidson & Janssens, 2006; Metcalfe *et al*., 2011). One such factor is vegetation, with shifts in plant community structure being reported in many biomes in response to climate change (Parmesan & Yohe, 2003; Elmendorf *et al*., 2012).

Vegetation is fundamental to terrestrial ecosystem carbon dynamics, being the source of photosynthetic carbon for the soil food web. It has been suggested that warming effects on plant growth and vegetation composition may drive greater uptake of atmospheric CO_2_, offsetting losses caused by ecosystem respiration (Qian *et al*., 2010; IPCC, 2013). However, ecosystem respiration has two components, autotrophic (plant) and heterotrophic (soil) respiration, that respond differently to climate and vegetation change (Dorrepaal *et al*., 2009; Hartley *et al*., 2012; Hicks Pries *et al*., 2013). An increase in plant respiration is usually tightly coupled to an accompanying increase in photosynthesis (Hicks Pries *et al*., 2013), resulting in faster CO_2_ turnover but no change in net ecosystem CO_2_ flux. Soil respiration, however, can increase independently of any compensatory responses in plant production (Hartley *et al*., 2012). Given that the Earth's soils represent carbon that has been fixed and stored over several millennia, soil respiration encompasses the degradation of organic compounds with ages spanning from minutes to centuries. A greater proportional contribution of ancient carbon to soil respiration could thus signal a long‐term loss of stable (Bosatta & Ågren, 1999), previously ‘locked‐up’, organic matter from soil, irrespective of net ecosystem CO_2_ flux (Dorrepaal *et al*., 2009; Hartley *et al*., 2012).

Northern peatlands are critical to the global carbon cycle, being the largest terrestrial organic carbon store and vulnerable to rapid temperature change (Dise, 2009; IPCC, 2013). Warming in these ecosystems has been shown to drive loss of ancient carbon from peat through ecosystem respiration (Dorrepaal *et al*., 2009). However, vegetation composition can additionally alter the response of peatland ecosystem respiration to warming, due to different vegetation types varying in productivity (Ward *et al*., 2013; Walker *et al*., 2015), root and litter inputs (Cornelissen *et al*., 2007; Ward *et al*., 2015) and plant–microbe associations (Read *et al*., 2004; Stępniewska & Goraj, 2014). Northern peatlands are dominated by four vegetation types, namely bryophytes, graminoids, dwarf‐shrubs and trees (not naturally present in UK peatlands) (Rodwell, 1991), which differ considerably in their ecophysiological traits. For example, *Sphagnum* moss species produce decay‐resistant litter that promotes low rates of soil respiration (Dorrepaal *et al*., 2005), but are expected to have limited influence at the ecosystem level due to their low productivity relative to dwarf‐shrubs and graminoids (Walker *et al*., 2015). By comparison, the ubiquitous graminoid *Eriophorum vaginatum* grows rapidly and generates litter that is decomposable (Trinder *et al*., 2008), leading to greater rates of decomposition and short‐term carbon turnover (Ward *et al*., 2009, 2015). Climate warming has been shown to increase ecosystem respiration relative to graminoid photosynthesis (Ward *et al*., 2013), suggesting that greater dominance of graminoids in peatlands could accelerate carbon loss and create a positive feedback to climate change. In contrast, the dominant UK dwarf‐shrub *Calluna vulgaris* suppresses activity throughout the soil food web (Ward *et al*., 2015) and reduces rates of soil respiration (Ward *et al*., 2009). While the mechanism explaining the inhibitory effect of *C. vulgaris* on microbial activity is currently unclear, warming has been shown to cause the largest increase in net ecosystem CO_2_ uptake when dwarf‐shrubs are present (Ward *et al*., 2013), suggesting that greater dwarf‐shrub growth in response to warming increases carbon sequestration. This is in agreement with observations that warming‐driven expansions of dwarf‐shrubs in arctic ecosystems increase net primary production (Qian *et al*., 2010; Pearson *et al*., 2013). However, vascular plant production has also been associated with priming in the arctic, leading to decomposition of ancient soil carbon (Hartley *et al*., 2012). Moreover, studies in northern peatlands have likewise shown that the presence of vegetation facilitates the liberation of ancient carbon from peat (Hardie *et al*., 2009). Ultimately, changes in the composition of vegetation have the potential to amplify or diminish warming effects on decomposition of ancient, previously ‘locked‐up’, organic matter from peat. Nevertheless, almost nothing is currently known about how changes in vegetation composition affect the source and age of peatland ecosystem respiration.

Numerous destructive methods exist for partitioning ecosystem respiration into component sources (e.g. root exclusion, girdling and trenching; Kuzyakov, 2006). However, all cause perturbations to the plant–soil system and none are able to explicitly determine CO_2_ age. Atomic bomb testing in the mid‐20th Century caused a pulse of radiocarbon in the atmosphere, known as the bomb‐^14^CO_2_ spike (Levin *et al*., 2010), which has been falling since then from a value of approximately 190 %Modern to a contemporary value of 103 %Modern. The bomb‐^14^CO_2_ spike can be used to estimate the contribution of recent carbon (<1 year since fixation; 103 %Modern), years‐ to decades‐old carbon (104 %Modern to 190 %Modern) and ancient carbon (e.g. centuries‐ to millennia‐old; below 100 %Modern) to respired CO_2_ (Hardie *et al*., 2009; Hartley *et al*., 2012; Hicks Pries *et al*., 2013). While ecosystem respiration represents carbon respired from a range of sources, radiocarbon measurements can be coupled with isotope mass balance approaches that use the flux and isotopic signature of ecosystem respiration to distinguish between plant and soil respiration (e.g. Hardie *et al*., 2009; Hartley *et al*., 2012). Together, these techniques represent a powerful tool for assessing warming and vegetation effects on the source of carbon respired from any ecosystem.

Here, we used an established peatland warming and vegetation manipulation experiment (Ward *et al*., 2013) coupled with *in situ* ^14^C measurements of ecosystem respiration to determine the effects of warming and different vegetation types on ancient peatland carbon release. Specifically, we tested the hypothesis that warming promotes the release of ancient, pre bomb‐^14^CO_2_ spike, carbon through ecosystem respiration and that its effects are modified by vegetation composition.

## Materials and methods

### Study site and experimental design

The experiment was located on a subarctic blanket peat site in northern England (55°64′N, 2°45′W; altitude 550 m). Mean annual temperature is 6.0 °C, and mean annual precipitation is 2016 mm (14 years average; UK Environmental Change Network). The vegetation community consists of three plant functional types, namely dwarf‐shrubs, graminoids and bryophytes. We established a fully factorial climate warming and vegetation removal experiment in 2009 (Ward *et al*., 2013). Vegetation manipulations were implemented by removing selected aboveground vegetation to create plots (1.5 m^2^) containing none (bare), all combinations of one or two plant functional types and a fully vegetated control. A warming treatment was added to half of the plots using passive open top chambers (Marion *et al*., 1997), generating ambient and elevated temperature versions of every vegetation treatment. For this study, we used ambient and elevated bare, single vegetation type and fully vegetated treatments from three replicate blocks. Ecosystem respiration and ^14^CO_2_ data were collected in July 2013 (*n* = 3), alongside associated measurements of water table height (manual readings from dipwells), air temperature in the vegetation canopy and soil temperature at 5 cm below the surface (Hobo Pendant loggers, Onset, UK). Air temperature and precipitation during this growing season were within 0.15 °C and 0.01 mm of the 2000–2013 average, respectively (Fig. S1). Additional measurements of ecosystem respiration taken during the 2009, 2010 and 2012 growing seasons also confirmed that 2013 measurements represented consistent interannual responses (Fig. S2).

### Ecosystem respiration flux measurements

Measurements of CO_2_ were taken by enclosing permanent airtight collars (*h* = 10 cm; *d* = 30 cm) installed at the surface‐peat interface with dark chambers (*h* = 35 cm). Ecosystem respiration flux was measured in July 2013 using an infrared gas analyser (2 min closure time; EGM‐4; PP Systems, Amesbury, MA, USA) (Ward *et al*., 2013) and determined using a linear regression approach that corrected for collar area, enclosure volume and air temperature (Gray *et al*., 2013; Ward *et al*., 2013).

### Radiocarbon sampling and analysis

Samples were collected for ^14^C analysis from the same chambers immediately after ecosystem respiration measurements using an established molecular sieve sampling system (Hardie *et al*., 2005; Hartley *et al*., 2012). Enclosed chambers were first scrubbed of atmospheric CO_2_ and left to allow build‐up of respired CO_2_. After CO_2_ accumulation (over 1000 ppm), chamber air was circulated through a system containing a zeolite molecular sieve cartridge (type 13X, 1.6 mm pellets; Sigma‐Aldrich, St. Louis, MO, USA) to capture CO_2_. Samples were returned to the NERC Radiocarbon Facility (East Kilbride, UK), where CO_2_ was thermally recovered (425 °C), cryogenically purified and split into aliquots. One aliquot was analysed for ^13^C/^12^C on a dual input isotope ratio mass spectrometer (Delta V; Thermo Fisher Scientific, Waltham, MA, USA), expressed as ‰ relative to the Vienna PDB standard. Another aliquot was concentrated onto a graphite target and analysed for ^14^C by accelerator mass spectrometry at the Scottish Universities Environmental Research Centre (SUERC, East Kilbride, UK). Following convention (Stuvier & Polach 2013), ^14^C data were normalised to −25‰ *δ* ^13^C to correct for mass‐dependent isotopic fractionation using:

(1) $$N=S\times \left(\frac{1+(-25÷10^{3})}{1+(\delta^{13}C_{S}÷10^{3})}\right)$$

where *N* is the normalised ^14^C/^13^C ratio of the sample, *S* is the raw ^14^C/^13^C ratio of the sample, and *δ* ^13^C_S_ is the ^13^C/^12^C ratio (‰) of the sample. Normalised data were expressed (%Modern) with reference to the activity of the NBS Oxalic Acid international radiocarbon standard using:

(2) $$\%\text{Modern}=\left(\frac{N}{O}\right)\times 100$$

where *O* is the ^14^C/^13^C ratio of the standard normalised to −19‰ *δ* ^13^C (Table S1).

To correct for any atmospheric CO_2_ that may have leaked into the chambers during sampling, we used *δ* ^13^C data to calculate the proportion of atmospheric CO_2_ in measured samples (Gaudinski *et al*., 2000):

(3) $$\text{Air}=\frac{\delta_{s}-\delta_{k}}{\delta_{a}-\delta_{k}}$$

where *δ*_s_ is the sample *δ* ^13^C value (‰), *δ*_a_ is the atmospheric *δ* ^13^C value (measured at −9‰ at time of sampling), and *δ*_k_ is the sample *δ* ^13^C value in the absence of any atmospheric contamination (‰). We derived *δ*_k_ using Keeling plots generated separately for different treatments (Fig. S3). Sample ^14^C contents were then corrected for atmospheric contamination using:

(4) $$\Delta_{\mathrm{cn}}=\frac{\Delta_{n}-\left(\text{Air}\times \Delta_{a}\right)}{\left(1-\mathrm{Air}\right)}$$

where Δ_cn_, Δ_n_ and Δ_a_ are the ^14^C contents (%Modern) of the corrected sample, uncorrected sample and atmosphere (measured at 103 %Modern at time of sampling), respectively (Gaudinski *et al*., 2000).

### Two‐component partitioning calculations

We used a two‐component isotope mass balance (Gaudinski *et al*., 2000; Hardie *et al*., 2009) to determine whether any vegetation type facilitated additional respiration from peat. Specifically, we described ecosystem respiration in different treatments as the product of peat respiration (i.e. ecosystem respiration in the absence of plants) vs. plant respiration (i.e. pure plant respiration plus additional peat respiration induced by the presence of plants):

(5) $$\left(\Delta_{e}\times f_{e}\right)=\left(\Delta_{p}\times f_{p}\right)+\left(\Delta_{s}\times f_{s}\right)$$

where Δ_p_, Δ_e_ and Δ_s_ are the ^14^C contents (%Modern) of plant respiration, ecosystem respiration and peat respiration, respectively, and *f*_p_, *f*_e_ and *f*_s_ are their fluxes (mg CO_2_–C m^−2^ h^−1^). We assumed that the ^14^C content and flux of bare treatment respiration represented that of peat respiration and that plant respiration flux could be calculated as:

(6) $$f_{p}=f_{e}-f_{s}$$

In doing so, we were able to derive the ^14^C content (age) of plant respiration as the only unknown in Eqn (4):

(7) $$\Delta_{p}=\frac{\left(\left(\Delta_{e}\times f_{e}\right)-\left(\Delta_{s}\times f_{s}\right)\right)}{f_{p}}$$

We expressed plant respiration ^14^C content both as %Modern and as a radiocarbon age [years BP, where 0 years BP = AD 1950 (Stuvier & Polach, 1977)], the latter based on the radioactive decay rate of ^14^C Eqn (3). Following convention, plant respiration ^14^C contents >100 %Modern were described as ‘modern’ (i.e. between AD1950 and present day).

(8) $$\text{years BP}=\frac{-8033\times \text{ln}(\Delta_{p}}{100)}$$

As autotrophs, plants respire carbon derived almost exclusively from recent photosynthesis, so pure plant respiration has a ^14^C content of approximately 103 %Modern (at the time of sampling; see Supplementary Information for supporting data). Any deviation of plant respiration ^14^C content away from this signature therefore represents dilution by an additional, older, source of respiration (i.e. plant‐induced peat respiration) and the magnitude of this deviation approximates the minimum mean age of the additional source.

Partitioning calculations were similarly performed on *δ* ^13^C data (Dorrepaal *et al*., 2009) to determine the *δ* ^13^C value of plant respiration in different treatments, using:

(9) $$\delta_{p}=\frac{\left(\left(\delta_{e}\times f_{e}\right)-\left(\delta_{s}\times f_{s}\right)\right)}{f_{p}}$$

where *δ*_p_, *δ*_e_ and *δ*_s_ are the *δ* ^13^C values (‰) of plant respiration, ecosystem respiration and peat respiration, respectively.

All partitioning calculations were performed at the treatment level (*n* = 3), using means for ^14^C content (Hardie *et al*., 2009) and data generated by Keeling plots for *δ* ^13^C to correct for atmospheric contamination (Fig. S3; Dorrepaal *et al*., 2009). Using this approach, we were able to characterise vegetation and warming effects on the presence, minimum age (^14^C content) and potential origin (*δ* ^13^C value; Dorrepaal *et al*., 2009; Billett *et al*., 2012) of plant‐induced peat respiration.

### Modelling plant‐induced peat respiration flux

Where plant‐induced peat respiration occurred, we estimated its potential absolute flux (mg CO_2_–C m^−2^ h^−1^) by expanding the two‐component mass balance approach to distinguish between pure plant respiration and plant‐induced peat respiration (Hardie *et al*., 2009):

(10) $$\left(\Delta_{e}\times f_{e}\right)=\left(\Delta_{\mathrm{pl}}\times f_{\mathrm{pl}}\right)+\left(\Delta_{i}\times f_{i}\right)+\left(\Delta_{s}\times f_{s}\right)$$

where Δ_pl_ and Δ_i_ are the ^14^C contents (%Modern) of pure plant respiration and plant‐induced peat respiration, respectively, and *f*_pl_ and *f*_i_ are their fluxes (mg CO_2_–C m^−2^ h^−1^). We assumed that the ^14^C content and flux of bare treatment respiration represented that of peat respiration, that the ^14^C content of pure plant respiration was 103 %Modern (see Supplementary Information), and that the fluxes of plant‐induced peat respiration and pure plant respiration could be calculated using Eqns (6) and (7), respectively.

(11) $$f_{i}=\left(\frac{f_{p}}{100}\right)\times a$$

(12) $$f_{\mathrm{pl}}=f_{p}-f_{i}$$

where *a* is the contribution (%) of plant‐induced peat respiration flux to plant respiration flux. Unique solutions were not possible due to the presence of too many unknowns, so we modelled scenarios where the contribution of plant‐induced peat respiration was between 10% and 50% of the plant respiration flux (10% intervals).

Through this, we derived a range of possible fluxes (mg CO_2_–C m^−2^ h^−1^) for plant‐induced peat respiration, which were considered plausible if corresponding ^14^C contents indicated a source of respiration that was fixed <5000 years BP (based on the approximate age of basal peat at the site; Billett *et al*., 2012).

### Statistical analysis

Linear mixed‐effects models were undertaken in R (R Development Core Team, Austria) using the package ‘nlme’ to test for effects of warming, vegetation type and their interaction on ecosystem respiration flux and ^14^C content. For ecosystem respiration flux, we included a random term for block, and for ^14^C content, we included random terms for block and sample temperature (mean of internal chamber temperature during enclosure; measured with Hobo Pendant Loggers, Onset, UK). In all cases, model assumptions were scrutinised using fitted values vs. residuals plots and QQ plots; where necessary, response variables were log_10_ transformed and models were refined to account for unequal variance between levels of explanatory variables (Zuur *et al*., 2010). Significance of fixed effects was determined using single term deletions coupled with likelihood ratio (LR) tests, retaining variables in models with *P* < 0.05.

To determine whether observed responses of ecosystem respiration ^14^C content occurred due to changes in microclimate, we used Pearson's product‐moment correlations to test for significant associations between ecosystem respiration ^14^C content (%Modern) and air temperature (°C), soil temperature (°C) and water table height (cm below surface) irrespective of experimental treatment. Finally, we used a Pearson product‐moment correlation to determine whether older modelled plant respiration ages (i.e. lower ^14^C content) were significantly associated with carbon from deeper in the peat profile (i.e. higher *δ* ^13^C value; Dorrepaal *et al*., 2009; Billett *et al*., 2012).

## Results

### Warming and vegetation effects on ecosystem respiration flux and ^14^C content

**Figure 1:**
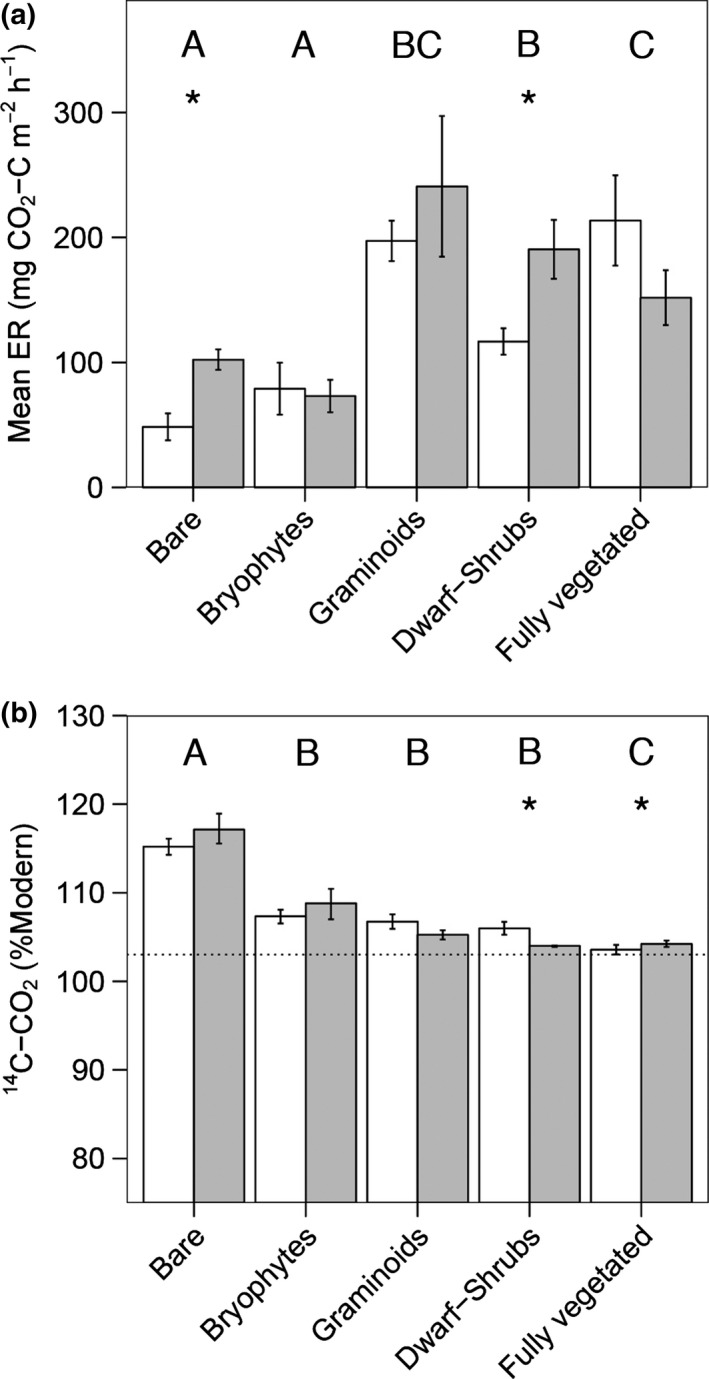
Warming and vegetation effects on the size and source of ecosystem respiration. Mean (±SE) ecosystem respiration (ER) (a) flux (mg CO_2_–C m^−2^ h^−1^) and (b) ^14^C content (%Modern) under different vegetation treatments and an ambient (white) or elevated (grey) warming treatment. Significant differences (*P* < 0.05) are shown by different letters for vegetation type and by a ‘*’ for warming. For (b), changes in ^14^C content towards that of the contemporary atmosphere (dotted line; 103 %Modern) could be driven by plant respiration (~103 %Modern) or by plants promoting mineralisation of ancient (<100 %Modern) peat carbon.

Ecosystem respiration flux (Fig. 1a) was greatest when either dwarf‐shrubs or graminoids were present (LR = 36.6, df = 4,12, *P* < 0.0001), being increased by 145% and 144% relative to the bare and bryophyte only treatments, respectively. By comparison, ecosystem respiration flux did not significantly differ between the bare and bryophyte only treatments. Warming significantly increased ecosystem respiration flux in the bare (by 111%) and dwarf‐shrub only (by 63%) treatments (LR = 12.3, df = 4,16, *P* = 0.0156), but had no effect in the bryophyte, graminoid only or fully vegetated treatments.

Ecosystem respiration ^14^C content (%Modern; Fig. 1b) was reduced in the presence of vegetation (LR = 37.1, df = 4,13, *P* < 0.0001). Warming decreased ecosystem respiration ^14^C content by 2 %Modern in the dwarf‐shrub only treatment and increased it by 1 %Modern in the fully vegetated treatment (LR = 15.8, df = 4,18, *P* = 0.0034). Warming did not affect ecosystem respiration ^14^C content in the bare, bryophyte only or graminoid only treatments. When considered irrespective of experimental treatment, we found that ecosystem respiration ^14^C content was not significantly associated with air temperature (*r* = 0.22, df = 24, *P* = 0.2704), soil temperature (*r* = 0.10, df = 17, *P* = 0.6694) or water table height (*r* = −0.05, df = 28, *P* = 0.7817). This means that warming had the greatest effect on peat ^14^C release *via* its influence on vegetation.

### Warming and vegetation effects on plant‐induced peat respiration

**Figure 2:**
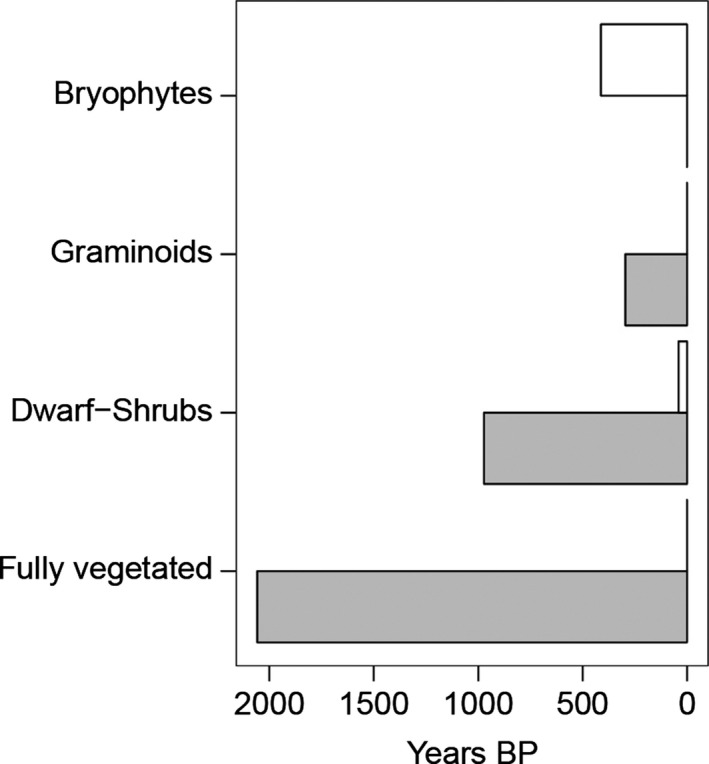
Warming and vegetation effects on ancient peatland carbon release. The modelled mean radiocarbon age of plant respiration (years BP) under different vegetation treatments and an ambient (white) or elevated (grey) warming treatment. Deviations from a modern signature indicate the presence of plant‐induced peat respiration, and the magnitude of this deviation approximates the mean minimum age of the additional carbon source. Bryophytes prevented any plant‐induced peat respiration at elevated temperature.

Two‐component partitioning calculations showed that modelled plant respiration deviated from a pure plant respiration signature (i.e. 103 %Modern) in all but the warmed bryophyte only treatment (Table 1), indicating that vegetation facilitated plant‐induced peat respiration in these treatments. At ambient temperature, the mean age of plant respiration only deviated considerably from a pure plant signature (i.e. 103 %Modern) in the bryophyte only treatment (Fig. 2). Specifically, plant respiration in the ambient bryophyte only treatment had a mean age of 412 years BP (94.8 %Modern), whereas in the ambient dwarf‐shrub only treatment it had a mean age of 40 years BP (99.5 %Modern) and was modern in the ambient graminoid only and fully vegetated treatments (104.0–101.5 %Modern, respectively).

**Table 1:** The modelled age (^14^C content) and potential source (*δ* ^13^C value) of combined plant and plant‐induced peat respiration

	^14^C content		*δ* ^13^C value	
	Ambient	Elevated	Ambient	Elevated
	%Modern	%Modern	‰	‰
Bryophytes	94.8	–a	−30.6	–a
Graminoids	104.0	96.4	−27.0	−21.1
Dwarf‐shrubs	99.5	88.6	−27.4	−21.0
Fully vegetated	101.5	77.4	−29.5	−15.2

a Bryophytes prevented any plant‐induced peat respiration at elevated temperature (Supplementary Methods).

Warming facilitated plant‐induced peat respiration when dwarf‐shrubs or graminoids were present, an effect not observed when only bryophytes were present (Table 1). Dwarf‐shrubs had a larger effect than graminoids, in that warming increased the mean age (Fig. 2) of plant respiration by approximately 900 years in the dwarf‐shrub only treatment (i.e. a reduction of 10.9 %Modern) and by approximately 300 years in the graminoid only treatment (i.e. a reduction of 7.6 %Modern). However, the strongest warming effect on the mean age of plant respiration was observed when both dwarf‐shrubs and graminoids were present in the fully vegetated treatment, where it increased by approximately 2100 years under warming (i.e. a reduction of 24.1 %Modern).

**Figure 3:**
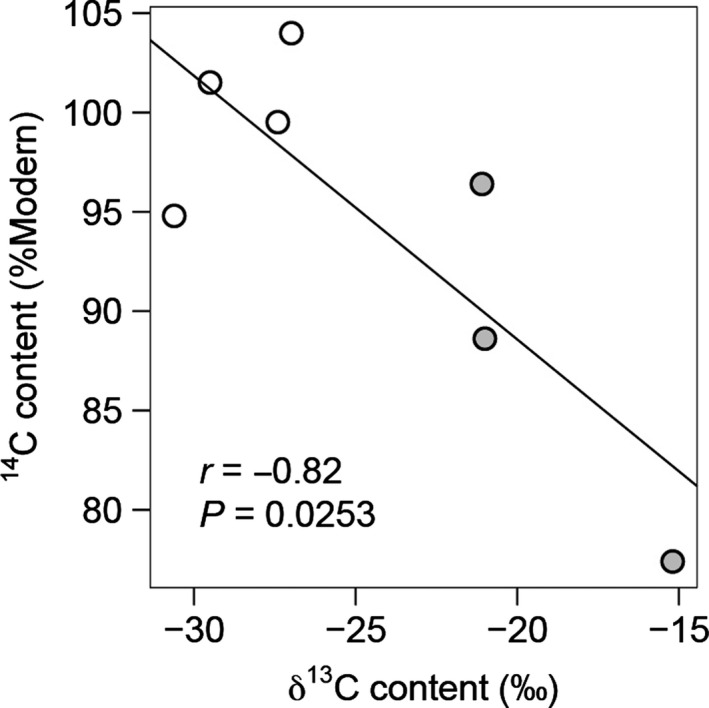
Relationship between the age and potential source of combined plant and plant‐induced respiration. Age (^14^C content; %Modern) and source (*δ* ^13^C value; ‰) were derived at the treatment level using a partitioning approach and are displayed as either ambient (white) or elevated (grey) temperature. There was a significant relationship between age and source (Pearson product‐moment correlation: *r* = −0.82, df = 5, *P* = 0.0253).

The mean *δ* ^13^C value of plant respiration did not strongly differ between vegetation types at ambient temperature (Table 1). However, warming increased the mean *δ* ^13^C value of plant respiration by 6.4‰ in the dwarf‐shrub only treatment and by 5.9‰ in the graminoid only treatment, and its effect was greatest in the fully vegetated treatment where it increased the mean *δ* ^13^C value of plant respiration by 14.3‰. We also found a significant negative correlation between the modelled ^14^C content (%Modern) and *δ* ^13^C value (‰) of plant respiration irrespective of experimental treatment (*r* = −0.82, df = 5, *P* = 0.0253), with warmed plots possessing lower ^14^C contents and higher *δ* ^13^C values (Fig. 3).

Three‐component partitioning calculations showed that modelled fluxes of plant‐induced peat respiration (Table 2) were lowest in the ambient bryophyte only treatment, ranging from 6.1 mg CO_2_–C m^−2^ h^−1^ to 15.3 mg CO_2_–C m^−2^ h^−1^ (assuming a 20–50% contribution to the total plant respiration flux, respectively). Modelled fluxes of plant‐induced peat respiration were highest when all vegetation types were present at between 16.5 mg CO_2_–C m^−2^ h^−1^ (10% contribution) and 82.6 mg CO_2_–C m^−2^ h^−1^ (50% contribution), but were also high in the graminoid only treatment at between 14.9 mg CO_2_–C m^−2^ h^−1^ (10% contribution) and 69.4 mg CO_2_–C m^−2^ h^−1^ (50% contribution).

**Table 2:** The modelled flux (mg CO_2_–C m^−2^ h^−1^) of plant‐induced peat respiration under scenarios where it represents 10–50% of the plant respiration flux

Contribution to flux (%)	Bryophytes		Graminoids		Dwarf‐shrubs		Fully vegetated	
	Ambient	Elevated^a^	Ambient	Elevated	Ambient	Elevated	Ambient	Elevated
10	–	n.a.	14.9	–	6.8	–	16.5	–
20	6.1	n.a.	29.8	27.7	13.7	–	33.0	–
30	9.2	n.a.	44.7	41.6	20.5	26.5	49.6	–
40	12.2	n.a.	59.5	55.5	27.3	35.3	66.1	–
50	15.3	n.a.	74.4	69.4	34.2	44.2	82.6	24.8

Missing values indicate scenarios in which modelled plant‐induced peat respiration ^14^C contents were implausible (i.e. >5000 years BP; Billett *et al*., 2012), and fluxes in parentheses indicate scenarios in which modelled plant‐induced peat respiration ^14^C contents were modern (i.e. >100 %Modern).

a Bryophytes prevented any plant‐induced peat respiration occurring at elevated temperature.

Warming increased the minimum proportional contribution of plant‐induced peat respiration to total plant respiration when vascular plants were present (Table 2), an effect not observed in the bryophyte only treatment. Specifically, the contribution of plant‐induced peat respiration increased from a minimum of 10% to 20% in the graminoid only treatment, from 10% to 30% in the dwarf‐shrub only treatment and from 10% to 50% in the fully vegetated treatment. Despite this, warming reduced modelled fluxes of plant‐induced peat respiration in all but the dwarf‐shrub only treatment, where they increased to between 26.5 mg CO_2_–C m^−2^ h^−1^ (30% contribution) and 44.2 mg CO_2_–C m^−2^ h^−1^ (50% contribution).

## Discussion

There is mounting concern that rapid warming in northern peatlands is causing liberation of ancient carbon from peat, raising questions about the future fate of the peatland carbon stock (Dorrepaal *et al*., 2009; Hicks Pries *et al*., 2013). In this study, we show that warming effects on the source of peatland ecosystem respiration are dependent on vegetation composition. We demonstrate that warming of approximately 1 °C triggers respiration of ancient peatland carbon when dwarf‐shrubs or graminoids are present and that this effect is negated when bryophytes are alone in the plant community. While measurements were taken on a single sampling date and hence must be interpreted with caution, both climate and CO_2_ fluxes during sampling were representative of 5‐year trends (Figs S1 and S2). This study consequently reveals that warming effects on ancient peatland carbon release vary with vegetation composition, and furthermore, its effects only occur in the presence of vascular plants. If consistent across peatland ecosystems, such plant‐induced peat respiration could represent a significant contribution to ecosystem respiration and a source of CO_2_ to the atmosphere that is currently not considered by the majority of global carbon cycle models.

We found that ecosystem respiration ^14^C content decreased in the presence of all vegetation types, with fully vegetated plots respiring CO_2_ with a ^14^C concentration most similar to that of the contemporary atmosphere. This confirms that the assimilation of modern photosynthetic carbon by the plant community directly influences the source of peatland ecosystem respiration. Further, warming only affected ecosystem respiration ^14^C content when dwarf‐shrubs were present (i.e. the dwarf‐shrub only and fully vegetated treatments), having no effect on the ^14^C content of bare peat respiration despite significantly raising CO_2_ efflux. Together, these findings show that dwarf‐shrubs, and to some extent graminoids, influence warming effects on the source of ecosystem respiration. At the same time, ecosystem respiration flux was greatest when either graminoids or dwarf‐shrubs were present, further illustrating the key role of vascular plants in regulating peatland CO_2_ fluxes (e.g. Ward *et al*., 2013). Our discovery is supported by 5 years of CO_2_ flux data from the same experiment (Fig. S2), suggesting that this is a long‐term response with no acclimation to either warming or vegetation change (Hartley *et al*., 2008; Dorrepaal *et al*., 2009). Two scenarios could explain the reduction in ecosystem respiration ^14^C content observed in vegetated treatments. First, vegetation may increase the proportional contribution of recently fixed carbon to ecosystem respiration, diluting its ^14^C content towards that of the contemporary atmosphere. This could occur *via* either greater plant respiration or enhanced mineralisation of recent root inputs by soil microbes. Under this scenario, vegetation would only affect the turnover of modern CO_2_, having no bearing on ancient carbon release. Second, vegetation may also prime microbial mineralisation of ancient carbon already present in peat (i.e. below 100 %Modern), the release of which would also dilute the ^14^C content of ecosystem respiration. Under this scenario, vegetation would facilitate ancient carbon release, with potential consequences for the fate of the peatland carbon stock.

Using mass balance approaches to distinguish between alternative scenarios, we found contrasting effects of bryophytes and vascular plants on the source of peatland ecosystem respiration. The presence of any vegetation induced additional peat respiration at ambient temperature. However, warming triggered respiration of ancient carbon exclusively when dwarf‐shrubs or graminoids (i.e. vascular plants) were present, halting it entirely in the bryophyte only treatment. Specifically, warming in vascular plant treatments increased both the mean age of plant‐induced peat respiration by up to 2100 years and its minimum proportional contribution to plant respiration by up to 40% (i.e. from 10% to 50% in the fully vegetated treatment; Table 2). Through this, we reveal that the occurrence of vascular plants facilitates warming‐driven liberation of ancient peatland carbon. Dwarf‐shrubs had the strongest effect, facilitating respiration with a mean age of approximately 1000–2100 years old under warming, potentially at a rate of between 25 and 44 mg CO_2_–C m^−2^ h^−1^. As both climate and CO_2_ fluxes during sampling were broadly representative of 5‐year trends (Figs S1 and S2), this suggests a considerable loss of ancient, possibly stable (Bosatta & Ågren, 1999), carbon from northern peatlands. Despite this, we found that absolute fluxes of ecosystem respiration on the day of measurement were unaffected by warming in the bryophyte, graminoid and fully vegetated treatments. Warming‐driven increases in the age of plant‐induced peat respiration were thus accompanied by declines in absolute fluxes of plant‐induced peat respiration in these treatments. This was most evident in the bryophyte only treatment, where warming reversed a small loss (6–15 mg CO_2_–C m^−2^ h^−1^) of approximately 400‐year‐old carbon that occurred in this treatment at ambient temperature. However, in real terms, ecosystem respiration was 1.5–3 times lower in the bryophyte only treatment than in any other vegetated treatment, further indicating that vascular plants have the greatest influence over ancient peatland carbon release. Indeed, warming in the dwarf‐shrub only treatment increased ecosystem respiration flux, resulting in a higher plant‐induced peat respiration flux (27–44 mg CO_2_–C^−2^ h^−1^) while also increasing its mean age by approximately 1000 years. Together, these findings indicate that vascular plants, and particularly dwarf‐shrubs, facilitate a greater contribution of ancient peatland carbon to ecosystem respiration under climate warming, albeit it at a lower absolute rate on this sampling date. Given that the long‐term sequestration of modern photosynthetic carbon as soil organic matter is far from certain (Conant *et al*., 2011), such a shift in the source of respired CO_2_ may signal the loss of a previously stable carbon pool.

Several mechanisms have been proposed to explain warming effects on peat, or soil respiration, reflecting both its direct action on belowground microclimate and its indirect action *via* changes to plant physiology (Davidson & Janssens, 2006; Fontaine *et al*., 2007; Dorrepaal *et al*., 2009; Metcalfe *et al*., 2011). Our results imply that vegetation is mostly responsible here because we found no correlations between ecosystem respiration ^14^C content and air temperature, soil temperature or water table height. This is further supported by our observation that warming had no effect on ecosystem respiration ^14^C content in the absence of vegetation. There is strong evidence that plants are able to prime organic matter decomposition (Fontaine *et al*., 2007; Hartley *et al*., 2012; Lindén *et al*., 2014), for instance by increasing microbial activity or intensifying nutrient competition within the soil food web. We suggest that priming occurs under warming when vascular plants are present and that this response is especially strong with dwarf‐shrubs due to associated mycorrhizae facilitating decomposition of recalcitrant, older (Bosatta & Ågren, 1999; Fontaine *et al*., 2007) carbon (Read *et al*., 2004). Bryophytes, as rootless organisms, cannot similarly prime decomposition and did not facilitate release of ancient carbon under warming in this study. The priming effects caused by vascular plants may even penetrate deep into the peat profile, for two reasons. First, plant‐induced peat respiration was at least twice as old as acrotelm (root‐zone) peat previously sampled from the same site (Hardie *et al*., 2007). Second, warming increased the modelled *δ* ^13^C value of plant respiration in vascular plant treatments, and we also found that older (^14^C‐depleted) respiration was significantly *δ* ^13^C‐enriched. This suggests that warming increases the contribution of deep peat carbon to ecosystem respiration in the presence of vascular plants (Dorrepaal *et al*., 2009; Billett *et al*., 2012). While *δ* ^13^C‐enriched respiration under rooting plants could alternatively be caused by transport of CO_2_ associated with methanogenesis (Stępniewska & Goraj, 2014), this is unlikely to be responsible here, since graminoids, which are key methane conduits (Gray *et al*., 2013), had weaker effects on ancient carbon release than dwarf‐shrubs.

While priming in mineral soils is well documented, there is currently no consensus on its occurrence in organic soils (e.g. Hartley *et al*., 2012; Lindén *et al*., 2014; Linkosalmi *et al*., 2015). Here, we present *in situ* evidence that vascular plants can prime decomposition of existing organic matter in peatlands, and moreover, they can also facilitate warming‐driven release of ancient carbon. Defining such persistent plant‐induced peat respiration as ‘priming’, however, should be carried out with caution, especially given that priming usually refers to pulses of respiration caused by episodic release of carbon into soil. Indeed, plant‐induced peat respiration in this study comprised a significant fraction of ecosystem respiration even in the fully vegetated treatment at ambient temperature (i.e. normal conditions). Regardless, it is apparent from these and other findings that vascular plants are key mediators of organic matter decomposition in many ecosystems, yet Earth System Models currently do not acknowledge any form of plant‐induced peat (or soil) respiration (Ostle *et al*., 2009; Lou *et al*., 2015). If such fluxes are universal across peatland and other subarctic and arctic ecosystems, we suggest that their incorporation into global carbon cycle models may greatly improve long‐term predictions of soil carbon stocks, and, through this, future climate change.

In conclusion, we show that climate warming in peatlands promotes ancient carbon release through ecosystem respiration and that this effect is facilitated by the presence of vascular plants. More work is now needed to determine the impacts of this discovery on the long‐term persistence of previously ‘locked‐up’ carbon in peatlands, particularly given previous findings that warming causes the greatest increase in net CO_2_ sink strength when dwarf‐shrubs are present in these shrub dominated ecosystems (Ward *et al*., 2013). Nevertheless, our findings have implications for feedbacks to the climate system due to both rising temperatures (IPCC, 2013) and the global significance of the peatland carbon stock (Dise, 2009). At the same time, vascular plant expansions are dominating vegetation change across many northern biomes (Elmendorf *et al*., 2012; Pearson *et al*., 2013). As such, this study raises questions about the fate of carbon stored not only in peatlands, but also in other high latitude ecosystems that have potential to feed back to climate change.

## Supporting information


**Figure S1.** Mean growing season (a) air temperature (°C ±SE) and (b) daily rainfall (mm ±1 SE) at the study site for the years 2000–2013, showing the 2000–2013 mean (solid line) and upper and lower bounds of one standard deviation (dashed lines). Vertical arrows indicate data relating to the 2013 study period. Data from the UK Environmental Change Network (www.data.ecn.ac.uk).
**Figure S2.** Mean (±SE) ER (mg CO_2_–C m^−2^ h^−1^) for the 2012 growing season (seven sampling dates; left panel) and the radiocarbon sampling date (July 2013; right panel) in the presence of different vegetation types and an ambient (blue) or elevated (grey) warming treatment.
**Figure S3.** Keeling plots used to determine ecosystem respiration *δ* ^13^C content (‰) in the absence of atmospheric contamination.
**Table S1.** Scottish Universities Environmental Research Centre (SUERC) publication codes and sample types.
**Table S2.** Photosynthetic tissue ^14^C (%Modern) and *δ* ^13^C content (‰) in different vegetation and warming treatments.
