# Supplementary material for: Vascular plants promote ancient peatland carbon loss with climate warming
Source: Glob Chang Biol. 2016 Mar 8;22(5):1880–9. doi: 10.1111/gcb.13213 (PMC4999049; doi:10.1111/gcb.13213)
Supplement: Supplementary file 1 — Figure S1. Mean growing season (a) air temperature (°C ±SE) and (b) daily rainfall (mm ±1 SE) at the study site for the years 2000–2013, showing the 2000–2013 mean (solid line) and upper and lower bounds of one standard deviation (dashed lines). Vertical arrows indicate data relating to the 2013 study period. Data from the UK Environmental Change Network (www.data.ecn.ac.uk). Figure S2. Mean (±SE) ER (mg CO2–C m−2 h−1) for the 2012 growing season (seven sampling dates; left panel) and the radiocarbon sampling date (July 2013; right panel) in the presence of different vegetation types and an ambient (blue) or elevated (grey) warming treatment. Figure S3. Keeling plots used to determine ecosystem respiration δ 13C content (‰) in the absence of atmospheric contamination. Table S1. Scottish Universities Environmental Research Centre (SUERC) publication codes and sample types. Table S2. Photosynthetic tissue 14C (%Modern) and δ 13C content (‰) in different vegetation and warming treatments. [file GCB-22-1880-s001.docx]

**Vascular plants trigger ancient peatland carbon loss under climate warming**

Tom N. Walker^1,2,3*^, Mark H. Garnett^4^, Susan E. Ward^3^, Simon Oakley^2^, Richard D. Bardgett^1^ and Nicholas J. Ostle^2,3^

**Supporting Information**

*Pure plant respiration ^14^C content*

We tested the assumption that autotrophic respiration would have a ^14^C content similar to that of the contemporary atmosphere (103 %Modern), recognising that plant-respired CO_2_ could originate from recently fixed photosynthate (approximately 103 %Modern) or hydrolysis of stored starch (potentially with an older ^14^C signature). Previous work on the same field site used a ^13^C pulse-labelling experiment to show that 96 % of ^13^C-CO_2_ assimilated by the plant community in the growing season is lost through respiration within 21 days (Ward *et al.* 2009). Plant respiration thus largely comprises recent photosynthetic carbon, with the remaining 4 % originating from both hydrolysis of starch in existing tissue and respiration of C assimilated after the ^13^C pulse. To determine the ^14^C content of existing tissue (as an indicator of the potential age of starch reserves), we took bulk photosynthetic tissue samples of each vegetation type from all measured plots (separated into ambient and elevated temperature treatments). These were washed in distilled water, oven dried (40 °C) and ground for ^14^C analysis. All samples were passed to the NERC Radiocarbon Facility (East Kilbride, Scotland), where they were combusted (900 °C) to recover tissue C as CO_2_ and analysed for ^14^C content as for CO_2_ samples (Methods). We used a mean of all three vegetation types to represent the tissue ^14^C content of fully vegetated treatments. In all cases, tissue ^14^C content deviated by less than ± 2 σ from the contemporary atmosphere ^14^C content (Supplementary Table S2). It is unlikely that respiration caused by starch hydrolysis is exclusively responsible for the remaining 4 % of unaccounted respiration. Nevertheless, we estimated the effect that this would have on overall plant respiration ^14^C content, assuming that starch C contributed to 4 % of the plant respiration flux:

(13) (1 × Δ_pl_) = (0.96 × Δ_r_) + (0.04 × Δ_s_)

Where Δ_pl_, Δ_r_ and Δ_s_ are the ^14^C contents (%Modern) of plant respiration, respiration of recent photosynthate (103 %Modern) and respiration caused by starch hydrolysis (i.e. bulk tissue ^14^C contents), respectively, and the constants represent their proportional contribution to the plant respiration flux. Under this scenario, plant respiration ^14^C contents varied between 102.98 %Modern (ambient graminoid treatment) and 103.04 %Modern (warmed dwarf-shrub treatment).

While the non-destructive nature of the experiment prevented sampling of belowground tissue, it has been suggested that root C can be older than the atmosphere (Sah *et al.* 2010). We therefore adjusted the above approach, substituting Δ_s_ with a value of 114.02 %Modern. In doing so, we tested a scenario whereby all remaining respiration originates exclusively from starch fixed and stored in roots 20 years prior to the sampling date (Levin *et al.* 2008), which again is unlikely. Under this scenario, overall plant respiration had a ^14^C content of 103.44 %Modern.

Together, this evidence indicates that autotrophic respiration generates CO_2_ with a ^14^C content that is not significantly different to the contemporary atmospheric CO_2_.

Table S1. Scottish Universities Environmental Research Centre (SUERC) publication codes and sample types.

| **Publication code** | **Sample type** | **^14^C content (%Modern ± 1 σ)** | **δ^13^C content (‰)** |
| --- | --- | --- | --- |
| SUERC-49395 | Carbon dioxide | 104.96 ± 0.48 | -21.6 |
| SUERC-49396 | Carbon dioxide | 107.22 ± 0.47 | -25.0 |
| SUERC-49397 | Carbon dioxide | 105.77 ± 0.49 | -25.9 |
| SUERC-49398 | Carbon dioxide | 104.01 ± 0.48 | -25.6 |
| SUERC-49399 | Carbon dioxide | 102.70 ± 0.47 | -21.8 |
| SUERC-49400 | Carbon dioxide | 103.65 ± 0.48 | -24.0 |
| SUERC-49401 | Carbon dioxide | 106.50 ± 0.49 | -26.6 |
| SUERC-49404 | Carbon dioxide | 107.16 ± 0.49 | -24.5 |
| SUERC-49405 | Carbon dioxide | 104.90 ± 0.46 | -24.7 |
| SUERC-49406 | Carbon dioxide | 103.64 ± 0.45 | -22.7 |
| SUERC-49407 | Carbon dioxide | 103.69 ± 0.45 | -24.7 |
| SUERC-49408 | Carbon dioxide | 103.83 ± 0.48 | -23.9 |
| SUERC-49409 | Carbon dioxide | 109.72 ± 0.50 | -23.2 |
| SUERC-49410 | Carbon dioxide | 112.45 ± 0.49 | -24.4 |
| SUERC-49411 | Carbon dioxide | 111.64 ± 0.51 | -25.1 |
| SUERC-49414 | Carbon dioxide | 113.91 ± 0.50 | -25.5 |
| SUERC-49415 | Carbon dioxide | 110.38 ± 0.51 | -25.9 |
| SUERC-49416 | Carbon dioxide | 112.82 ± 0.52 | -26.8 |
| SUERC-49417 | Carbon dioxide | 104.34 ± 0.48 | -22.5 |
| SUERC-49418 | Carbon dioxide | 107.59 ± 0.49 | -24.2 |
| SUERC-49419 | Carbon dioxide | 106.08 ± 0.46 | -25.9 |
| SUERC-49420 | Carbon dioxide | 104.17 ± 0.45 | -24.4 |
| SUERC-49421 | Carbon dioxide | 105.65 ± 0.48 | -23.1 |
| SUERC-49424 | Carbon dioxide | 103.57 ± 0.45 | -24.4 |
| SUERC-49425 | Carbon dioxide | 103.60 ± 0.45 | -19.6 |
| SUERC-49426 | Carbon dioxide | 104.47 ± 0.48 | -24.0 |
| SUERC-49427 | Carbon dioxide | 104.94 ± 0.47 | -22.5 |
| SUERC-49428 | Carbon dioxide | 105.54 ± 0.46 | -24.5 |
| SUERC-49429 | Carbon dioxide | 104.05 ± 0.48 | -24.6 |
| SUERC-49430 | Carbon dioxide | 108.86 ± 0.50 | -26.5 |
| SUERC-49434 | Plant tissue | 103.7 ± 0.45 | -27.8 |
| SUERC-49435 | Plant tissue | 102.8 ± 0.47 | -28.6 |
| SUERC-49436 | Plant tissue | 103.0 ± 0.47 | -27.9 |
| SUERC-49437 | Plant tissue | 103.9 ± 0.48 | -28.5 |
| SUERC-49438 | Plant tissue | 102.4 ± 0.45 | -26.0 |
| SUERC-49439 | Plant tissue | 102.8 ± 0.45 | -26.9 |

Table S2. Photosynthetic tissue ^14^C (%Modern) and δ^13^C content (‰) in different vegetation and warming treatments.

| **Warming** | **Vegetation type** | **Tissue ^14^C content (%Modern ± 1 σ)** | **Tissue δ^13^C content (‰)** |
| --- | --- | --- | --- |
| Ambient | Bryophytes | 103.7 ± 0.45 | -27.8 |
| Ambient | Graminoids | 102.4 ± 0.45 | -26.0 |
| Ambient | Dwarf-Shrubs | 103.0 ± 0.47 | -27.9 |
| Ambient | Fully vegetated | 103.0 (± 0.38)^†^ | -27.2 (± 0.62) ^†^ |
| Elevated | Bryophytes | 102.8 ± 0.47 | -28.6 |
| Elevated | Graminoids | 102.8 ± 0.45 | -26.9 |
| Elevated | Dwarf-Shrubs | 103.9 ± 0.48 | -28.5 |
| Elevated | Fully vegetated | 103.2 (± 0.37) ^†^ | -28.0 (± 0.56) ^†^ |

^†^ Fully vegetated data are means of other vegetation types (error term in parentheses represents ± 1SE)

Fig. S1. Mean growing season (a) air temperature (°C ± SE) and (b) daily rainfall (mm ± 1 SE) at the study site for the years 2000 to 2013, showing the 2000-2013 mean (solid line) and upper and lower bounds of one standard deviation (dashed lines). Vertical arrows indicate data relating to the 2013 study period. Data from the UK Environmental Change Network (www.data.ecn.ac.uk).

**
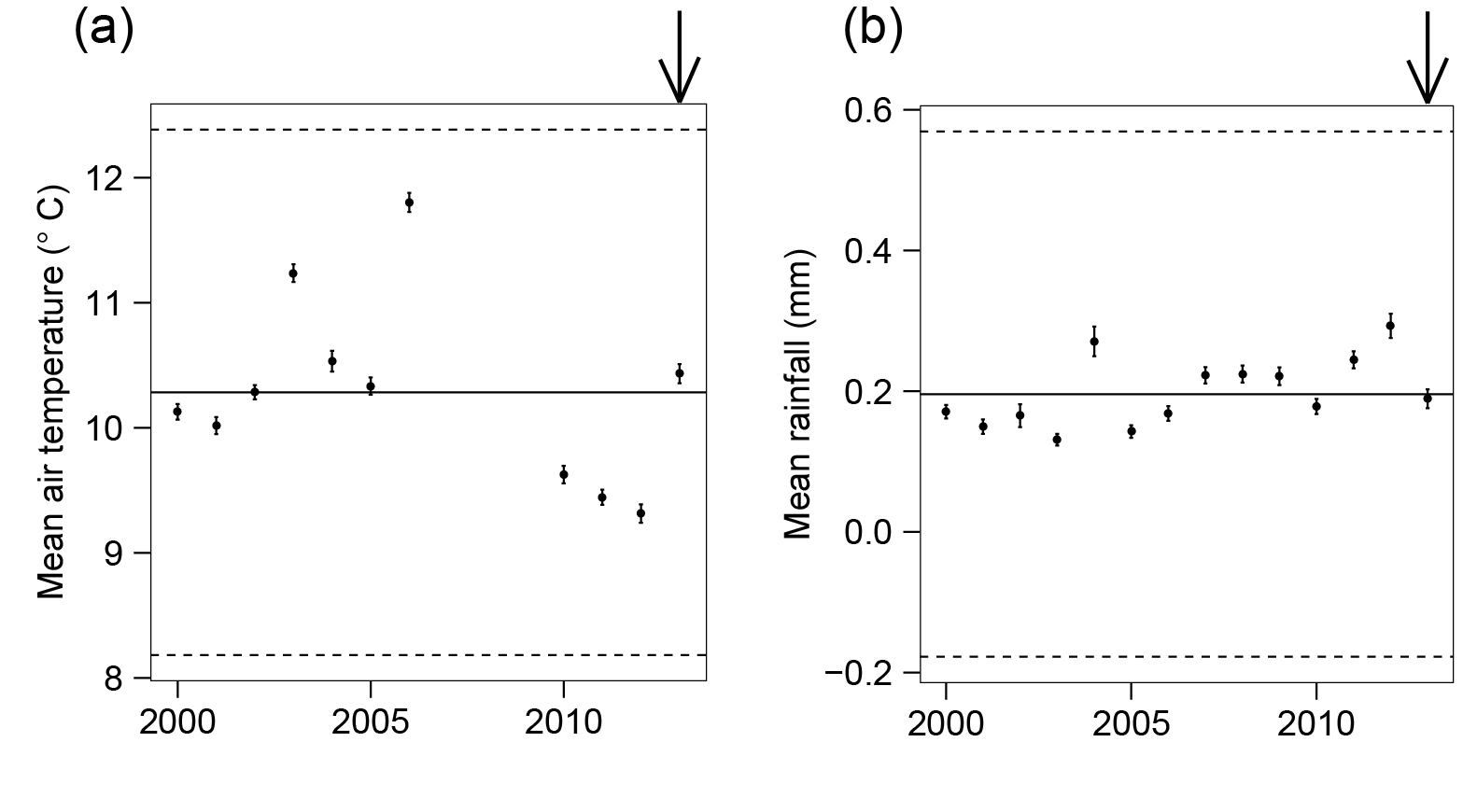
**

Fig. S2. Mean (± SE) ER (mg CO_2_-C m^-2^ h^-1^) for the 2012 growing season (7 sampling dates; left panel) and the radiocarbon sampling date (July 2013; right panel) in the presence of different vegetation types and an ambient (blue) or elevated (grey) warming treatment. Data from both years were consistent with measurements taken from the same experiment during the 2009 and 2010 growing seasons.

**
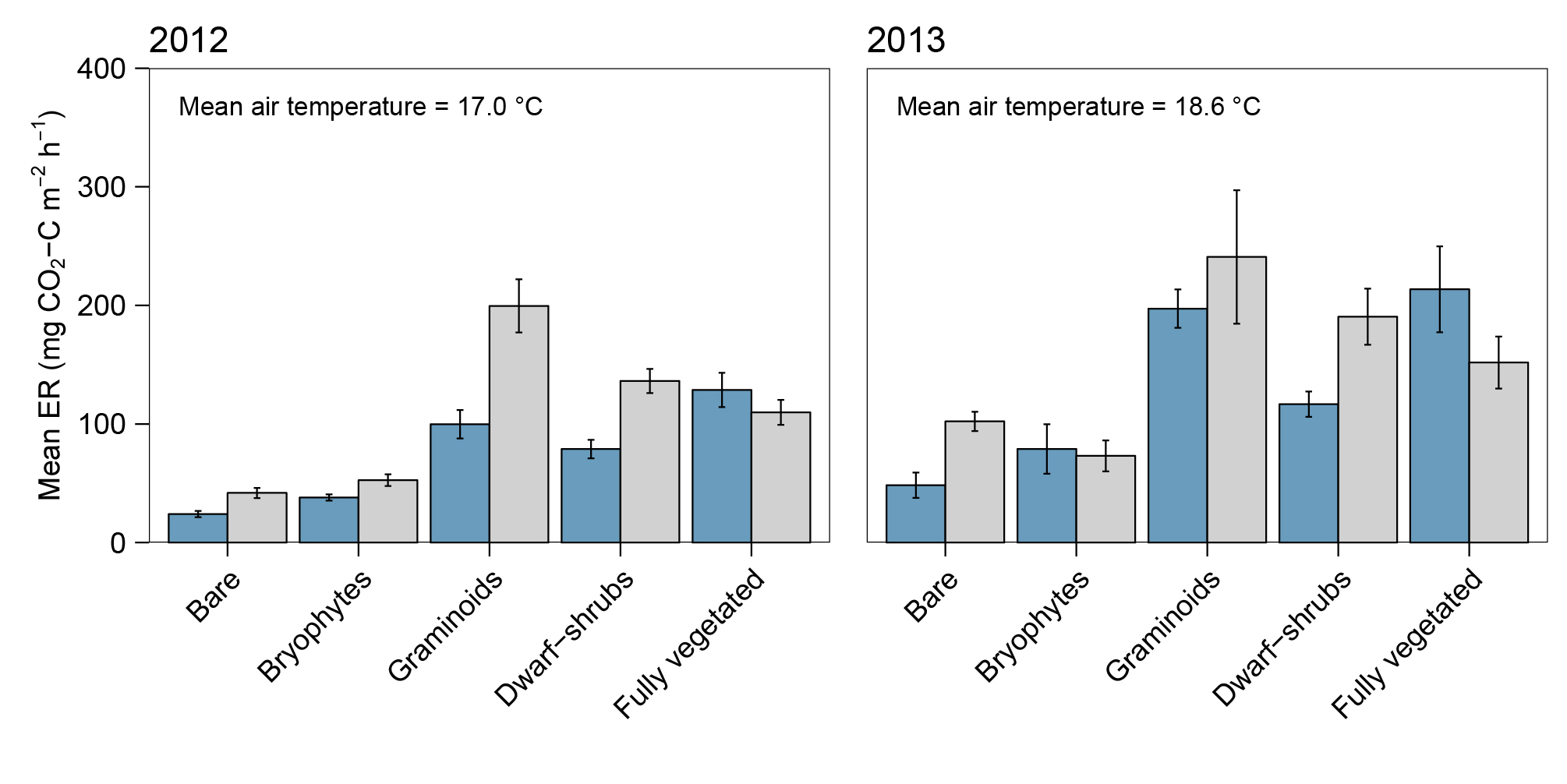
**

**Fig. S3. Keeling plots used to determine ecosystem respiration δ^13^C content (‰) in the absence of atmospheric contamination.** Data were collected from all measured plots on two sampling occasions (July 2010 and July 2013) and were analysed at the treatment level (i.e. different vegetation types at either ambient (blue; top panel) or elevated (grey; bottom panel) temperature) to provide sufficient replication (n = 9). Regressions between δ^13^C content (‰) and 1/CO_2_ (PPM^-1^) were used to determine the δ^13^C content (‰) of ecosystem respiration at CO_2_ = 0 PPM (i.e. y-intercept; r^2^ and *P*-values are also shown on each panel).


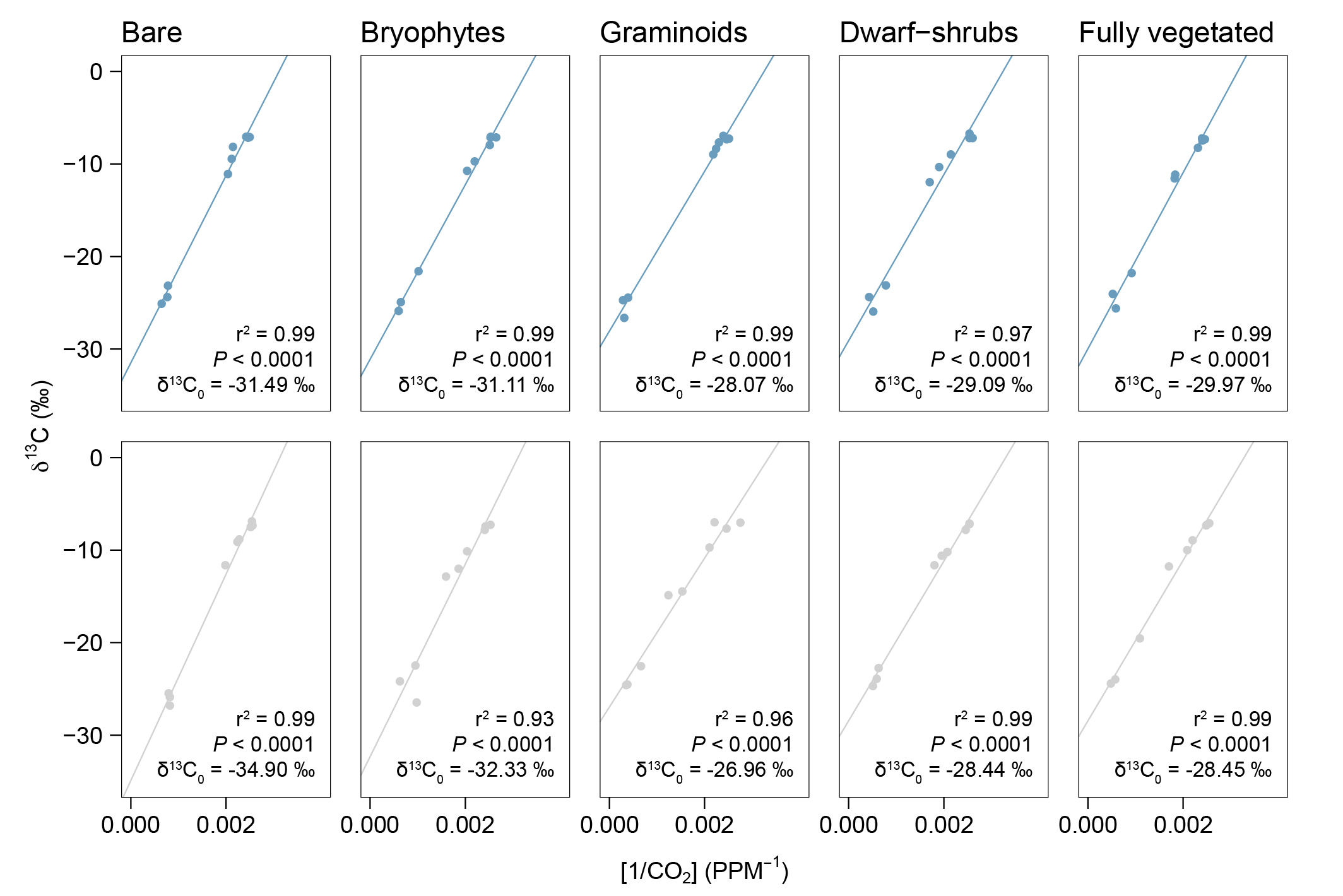


**Additional References**

Sah, S. P., H. Junger, M. Oinonen, M. Kukkola & H.-S. Helmisaari (2010): Does the age of fine root carbon indicate the age of fine roots in boreal forests? *Biogeochemistry* **104**: 91-102.
